# Economic Appraisal of Ontario's Universal Influenza Immunization Program: A Cost-Utility Analysis

**DOI:** 10.1371/journal.pmed.1000256

**Published:** 2010-04-06

**Authors:** Beate Sander, Jeffrey C. Kwong, Chris T. Bauch, Andreas Maetzel, Allison McGeer, Janet M. Raboud, Murray Krahn

**Affiliations:** 1 Department of Health Policy, Management and Evaluation, University of Toronto, Toronto, Canada; 2 Division of Clinical Decision-Making and Health Care Research, University Health Network, Toronto, Canada; 3 Toronto Health Economics and Technology Assessment Collaborative, Toronto, Canada; 4 Institute for Clinical Evaluative Sciences, Toronto, Canada; 5 Department of Family and Community Medicine, University of Toronto, Toronto, Canada; 6 Dalla Lana School of Public Health, University of Toronto, Toronto, Canada; 7 Department of Mathematics and Statistics, University of Guelph, Guelph, Canada; 8 Amgen (Europe) GmbH, Zug, Switzerland; 9 Division of Infectious Diseases, University Health Network, Toronto, Canada; 10 Faculty of Pharmacy, University of Toronto, Toronto, Canada; 11 Department of Medicine, University of Toronto, Toronto, Canada; Harvard School of Public Health, United States of America

## Abstract

**Background:**

In July 2000, the province of Ontario, Canada, initiated a universal influenza immunization program (UIIP) to provide free seasonal influenza vaccines for the entire population. This is the first large-scale program of its kind worldwide. The objective of this study was to conduct an economic appraisal of Ontario's UIIP compared to a targeted influenza immunization program (TIIP).

**Methods and Findings:**

A cost-utility analysis using Ontario health administrative data was performed. The study was informed by a companion ecological study comparing physician visits, emergency department visits, hospitalizations, and deaths between 1997 and 2004 in Ontario and nine other Canadian provinces offering targeted immunization programs. The relative change estimates from pre-2000 to post-2000 as observed in other provinces were applied to pre-UIIP Ontario event rates to calculate the expected number of events had Ontario continued to offer targeted immunization. Main outcome measures were quality-adjusted life years (QALYs), costs in 2006 Canadian dollars, and incremental cost-utility ratios (incremental cost per QALY gained). Program and other costs were drawn from Ontario sources. Utility weights were obtained from the literature. The incremental cost of the program per QALY gained was calculated from the health care payer perspective. Ontario's UIIP costs approximately twice as much as a targeted program but reduces influenza cases by 61% and mortality by 28%, saving an estimated 1,134 QALYs per season overall. Reducing influenza cases decreases health care services cost by 52%. Most cost savings can be attributed to hospitalizations avoided. The incremental cost-effectiveness ratio is Can$10,797/QALY gained. Results are most sensitive to immunization cost and number of deaths averted.

**Conclusions:**

Universal immunization against seasonal influenza was estimated to be an economically attractive intervention.

*Please see later in the article for the Editors' Summary*

## Abstract 2

Beate Sander and colleagues assess the cost-effectiveness of the program that provides free seasonal influenza vaccines to the entire population of Ontario, Canada.

## Editors' Summary

**Background:**

Annual outbreaks (epidemics) of influenza—a viral disease of the nose, throat, and airways—make millions of people ill and kill about 500,000 individuals every year. In doing so, they impose a considerable economic burden on society in terms of health care costs and lost productivity. Influenza epidemics occur because small but frequent changes in the viral proteins to which the immune system responds mean that an immune response produced one year by exposure to an influenza virus provides only partial protection against influenza the next year. Annual immunization with a vaccine that contains killed influenza viruses of the major circulating strains can boost this natural immunity and greatly reduce a person's chances of catching influenza. Consequently, many countries run seasonal influenza vaccine programs. These programs usually target people at high risk of complications from influenza and individuals likely to come into close contact with them, and people who provide essential community services. So, for example, in most Canadian provinces, targeted influenza immunization programs (TIIPs) offer free influenza vaccinations to people aged 65 years or older, to people with chronic medical conditions, and to health care workers.

**Why Was This Study Done?:**

Some experts argue, however, that universal vaccination might provide populations with better protection from influenza. In 2000, the province of Ontario in Canada decided, therefore, to introduce a universal influenza immunization program (UIIP) to provide free influenza vaccination to everyone older than 6 months, the first large program of this kind in the world. A study published in 2008 showed that, following the introduction of the UIIP, vaccination rates in Ontario increased more than in other Canadian provinces. In addition, deaths from influenza and influenza-related use of health care facilities decreased more in Ontario than in provinces that continued to offer a TIIP. But is universal influenza vaccination good value for money? In this study, the researchers evaluate the cost-effectiveness of the Ontario UIIP by comparing the health outcomes and costs associated with its introduction with the health outcomes and costs associated with a hypothetical continuation of targeted influenza immunization.

**What Did the Researchers Do and Find?:**

The researchers used data on TIIP and UIIP vaccine uptake, physician visits, emergency department visits, hospitalizations for influenza, and deaths from influenza between 1997 and 2004 in Ontario and in nine Canadian states offering TIIPs, and Ontario cost data, in their “cost-utility” analysis. This type of analysis estimates the additional cost required to generate a year of perfect health (a quality-adjusted life-year or QALY) through the introduction of an intervention. QALYs are calculated by multiplying the time spent in a certain health state by a measure of the quality of that health state. The researchers report that the cost of Ontario's UIIP was about twice as much as the cost of a TIIP for the province. However, the introduction of the UIIP reduced the number of influenza cases by nearly two-thirds and reduced deaths from influenza by more than a quarter compared with what would have been expected had the province continued to offer a TIIP, an overall saving of 1,134 QALYs. Furthermore, the reduction in influenza cases halved influenza-related health care costs, mainly because of reductions in hospitalization. Overall, this means that the additional cost to Ontario of saving one QALY through the introduction of the UIIP was Can$10,797, an “incremental cost-effectiveness ratio” of $10,797 per QALY gained.

**What Do These Findings Mean?:**

In Canada, an intervention is considered cost-effective from the point of view of a health care purchaser if it costs less than Canadian $50,000 to gain one QALY. These findings indicate, therefore, that for Ontario the introduction of the UIIP is economically attractive. Indeed, the researchers calculate that even if the costs of the UIIP were to double, the additional cost of saving one QALY by introducing universal immunization would remain below $50,000. Other “sensitivity” analyses undertaken by the researchers also indicate that universal immunization is likely to be effective and cost-effective in Ontario if other key assumptions and/or data included in the calculations are varied within reasonable limits. Given these findings, the researchers suggest that a UIIP might be an appealing intervention in other Canadian provinces and in other high-income countries where influenza transmission and health-care costs are broadly similar to those in Ontario.

**Additional Information:**

Please access these Web sites via the online version of this summary at http://dx.doi.org/10.1371/journal.pmed.1000256.

- A *PLoS Medicine* Research Article by Kwong and colleagues describes how the introduction of universal influenza immunization in Ontario altered influenza-related health care use and deaths in the province
- Wikipedia pages are available on QALYs and on cost-utility analysis (note that Wikipedia is a free online encyclopedia that anyone can edit; available in several languages)
- Bandolier, an independent online journal about evidence-based health-care, provides information about QALYs and their use in cost-utility analysis
- The UK National Institute for Health and Clinical Excellence has a webpage on Measuring effectiveness and cost-effectiveness: the QALY

## Introduction

Worldwide, annual epidemics of influenza cause significant morbidity and mortality and impose a considerable economic burden on society in terms of health care costs and lost productivity [1].

Seasonal prophylaxis with vaccines is the cornerstone of influenza management. Influenza vaccines are generally safe and effective. They reduce serologically confirmed influenza cases by 73% in healthy adults [2] and 58% in the elderly [3]. Influenza vaccination in young children decreases the incidence of influenza infection [4], acute otitis media [5],[6], and daycare absenteeism [7].

In most jurisdictions, seasonal prophylaxis with vaccines is currently recommended for people at high risk of complications, those capable of transmitting influenza to individuals at high risk of complications, and those who provide essential community services [8]. Nevertheless, vaccine coverage rates among high-risk groups in Canada have been low in the past, substantially lower than the target coverage rate (70%) set by a national consensus conference on influenza in 1993 [9]. In response to these findings, most provinces and territories in Canada offer free influenza vaccinations for those 65 y or older, those with chronic medical conditions, and health care workers (Targeted Influenza Immunization Program, TIIP) [10]. The age-based recommendations in the United States are more broad, including individuals 50 y or older and children aged 6 mo to 18 y [11].

In 2000, the province of Ontario, Canada, initiated a universal influenza immunization program (UIIP) [12] to provide free influenza vaccines for the entire population (6 mo of age or older). Introduction of this program was associated with greater overall increases in influenza vaccination rates, particularly in children and working-age adults, and greater reductions in influenza-associated mortality and health care use in Ontario compared to other provinces that maintained targeted programs [13],[14].

The cost-effectiveness of universal vaccination has not been evaluated, despite the investment of substantial financial resources. The objective of this study was to conduct an economic appraisal of Ontario's UIIP.

## Methods

An economic evaluation was performed to estimate health outcomes and costs related to universal vaccination compared to the hypothetical continuation of a targeted program for the entire population of Ontario (12.16 million).

### Data

All the input data are age specific (Tables S1 and S2) and used as such in the calculations. The seven age groups are 4 y and under, 5–19 y, 20–49 y, 50–64 y, 65–74 y, 75–84 y, and 85 y and older. Most results are aggregated and reported for the total program; supplementary information is available by age group (Table S3).

### Effectiveness of UIIP

This economic evaluation was informed by an earlier epidemiological study that compared outcomes in Ontario before and after universal immunization, using other provinces as controls [13]. Introduction of Ontario's UIIP was associated with greater overall increases in influenza vaccination rates in Ontario (from 18% in 1996 to 42% in 2005) compared to other provinces (from 13% to 28%). Therefore, there was a 9-percentage-point incremental increase in Ontario, and the relative increases were most evident among those under 65 y. A different pattern was observed among the elderly; for those aged 75 y or older, there were greater relative increases among other provinces compared to Ontario, although Ontario still maintained higher rates at all times.

The impact of Ontario's UIIP on influenza-associated mortality, hospitalizations, and visits to emergency departments (EDs) and doctors' offices was estimated using data from 1997 to 2004 (3 y before and 4 y after UIIP implementation). To estimate influenza-associated outcomes, we used multivariate regression models to predict events with influenza viral activity in the model, and we subsequently removed the influenza terms (i.e., viral surveillance data) from the model to generate a baseline function that represented the hypothetical absence of influenza. To calculate influenza-associated events, we subtracted the expected baseline events from observed events during periods of influenza activity. The models controlled for age, sex, province, influenza surveillance data, and temporal trends. Influenza-associated event rates in the overall population decreased 40%–60% more in Ontario than in other provinces for all study outcomes. The relative reductions were particularly prominent among age groups younger than 65, consistent with the changes in vaccine uptake. However, despite greater increases in vaccination rates among the elderly in other provinces compared to Ontario, the reductions in influenza-associated events among the elderly were either less pronounced or the same in Ontario compared to other provinces. The robustness of these findings was confirmed by numerous sensitivity analyses [13]. For a more detailed description of the regression model and results, see Text S1 and Table S6.

Side effects due to influenza vaccine were not included as they are usually mild. While there is a small risk of hospitalization because of Guillain-Barré syndrome (GBS), a population-based study found no statistically significant increase in hospital admissions because of GBS after introduction of UIIP [15].

### Resource Use: Physician Services and Hospitalizations

For the economic evaluation, the mean number of events per season post-UIIP implementation as observed in Ontario was compared with the expected number of events under hypothetical continuation of TIIP (Table 1; more detailed information can be found in Tables S1 and S2). To calculate the expected number of events under hypothetical continuation of TIIP for Ontario, the relative change of mean number of events pre-2000 (1997/1998 to 1999/2000) to post-2000 (2000/2001 to 2003/2004) observed in other Canadian provinces was applied to the pre-UIIP event rates in Ontario. This assumes that in Ontario vaccination coverage rates would have increased and influenza-related events decreased by the same relative magnitude as in other provinces after 2000.

**Table 1:** Mean annual influenza-related event rates and relative rates comparing post-UIIP to pre-UIIP event rates.

Event	Number of Events per 100,000 Population in Ontario, Mean (95% CI)		Post- vs. Pre-2000 Relative Rate, Mean (95% CI)	
	Pre-UIIP	Post-UIIP	Ontario	Other Provinces Combined
Office visits	813.58 (807.90; 819.20)	173.00 (169.90; 176.10)	0.21 (0.21; 0.22)	0.52 (0.51; 0.53)
ED visits	139.57 (137.50; 141.70)	43.57 (42.20; 44.90)	0.31 (0.30; 0.32)	0.69 (0.67; 0.70)
Hospitalizations	33.38 (32.20; 34.60)	8.49 (7.70; 9.30)	0.25 (0.23; 0.28)	0.44 (0.42; 0.46)
Deaths	12.00 (11.05; 12.96)	2.79 (2.15; 3.42)	0.23 (0.18; 0.30)	0.33 (0.28; 0.39)

Source: Kwong [13].

CI, confidence interval; ED, emergency department; UIIP, universal influenza immunization program.

All data were obtained for seven influenza seasons covering the years from 1997 to 2004 [13]. Physician services data were extracted from provincial datasets for Ontario (Ontario Health Insurance Plan, OHIP) and three other Canadian provinces (Quebec's Physician Claims Dataset, Alberta's Health Care Insurance Plan Dataset, and Manitoba's Medical Claims Dataset). Hospitalization data were obtained from Statistics Canada's Hospital Morbidity Database, a national hospital discharge dataset. Influenza-related events were identified using ICD 9/10 (International Statistical Classification of Diseases and Related Health Problems) service codes associated with pneumonia or influenza. These are complete datasets, covering all residents of the respective provinces.

Influenza-associated events decreased more in Ontario than other provinces: 75% versus 56% for hospitalizations, 69% versus 31% for ED use, and 79% versus 48% for doctors' office visits [13].

### Quality-Adjusted Life Years (QALYs) Lost from Influenza

The administrative data used to estimate resource use do not yield a count of influenza cases. The number of cases of influenza requiring health care was estimated using the number of office visits and ED visits and excluding repeat visits by the same patient for the same diagnosis within a 21-d window following an initial visit. According to this approximation, 90% of all influenza-associated office visits, 85% of all influenza-associated ED visits, and all influenza-associated hospitalizations were deemed to be discrete new cases of influenza. The remainder of visits were considered to be repeat visits. Multiple visits across settings by the same patient were not removed since the number of those was expected to be small (for example, only 0.07% of all health care contacts were hospitalizations, some of which may have had an office and/or ED visit before being hospitalized). It was assumed that patients who die due to influenza had at least one health care contact before death. Therefore, these were also not separately counted as cases as they were presumed to have been accounted for.

Quality weights as reported by Turner [16] were used to estimate QALYs lost per case of influenza due to morbidity. In this recent assessment by the National Institute for Health and Clinical Excellence (NICE) of antiviral treatment of influenza, utilities for influenza were estimated from patient health state valuations reported daily for 21 d in oseltamivir clinical trials. Utilities were generated by recalibrating the Likert scores as obtained in the clinical trials to mean visual analogue scale scores, which were then transformed to time trade-off scores. Quality of life data were available for “otherwise healthy adults,” “adults with co-morbidities,” and the “elderly.” In the absence of utility data for children, it was assumed that children have similar utility weights as “otherwise healthy adults” as both groups are similar in other health outcome measures, such as time to return to normal activity [17]. The utility weight for adults with co-morbidities as obtained in the clinical trials was applied to the 18.8% of the Ontario population aged 12 to 64 y deemed high risk because of chronic conditions [18]. Patients in the trials were recruited from Europe and North America. We assume that the clinical trial population is sufficiently similar to the Ontario population (high income Western countries with similar life expectancy and population health status) so that we can apply the utility estimates to the population under consideration here. The QALY gain per influenza case prevented (Table 2) was calculated by multiplying the duration of the symptomatic period by the utility decrement associated with influenza-related illness.

**Table 2:** Key data used in the economic evaluation.

Parameter	Base Case Analysis, Mean	Deterministic Sensitivity Analysis	
		Lower Confidence Limit (Worst Case)	Upper Confidence Limit (Best Case)
**Per Influenza Case Prevented**			
0–4 y	0.0146	0.0065	0.0146
5–19 y	0.0146	0.0065	0.0146
20–49 y	0.0174	0.0097	0.0245
50–64 y	0.0174	0.0044	0.0245
65–74 y	0.0293	0.0233	0.0349
75–84 y	0.0293	0.0233	0.0349
85+ y	0.0293	0.0233	0.0349
**Per Death Prevented**			
** Undiscounted**			
0–49 y	62	N/A	N/A
50–64 y	21	N/A	N/A
65–74 y	11	N/A	N/A
75–84 y	4	N/A	N/A
85+ y	1	N/A	N/A
** Discounted 3%**			
0–49 y	27	N/A	N/A
50–64 y	14	N/A	N/A
65–74 y	8	N/A	N/A
75–84 y	4	N/A	N/A
85+ y	1	N/A	N/A
** Discounted 5%**			
0–49 y	18	N/A	N/A
50–64 y	12	N/A	N/A
65–74 y	7	N/A	N/A
75–84 y	4	N/A	N/A
85+ y	1	N/A	N/A
**Unit Cost**			
Office visit	$35	$18	$60
ED visit	$220	$183	$371
Hospitalization	$6,418	$2,075	$21,548
TIIP	$19,946,556	$19,333,519	$20,944,646
UIIP	$40,000,000	$40,000,000	$40,000,000

Source: quality of life with influenza: Turner [16]; life expectancy: Statistics Canada [19]; quality of life adjustment by age in the absence of influenza: Mittmann [20]; office visits, ED visits, hospitalizations: Ontario Health Insurance Plan (OHIP) dataset; TIIP: calculated based on influenza immunization program cost before implementation of UIIP, vaccine coverage rates in Ontario pre-UIIP, and relative increase of influenza immunization coverage in other provinces [13],[56] (Nancy Peroff-Johnston, MOHLTC, personal communication, May 11, 2007); UIIP: Ministry of Health and Long-Term Care [56] (Nancy Peroff-Johnston, MOHLTC, personal communication, May 11, 2007).

ED, emergency department; TIIP, targeted influenza immunization program; UIIP, universal influenza immunization program.

To calculate QALYs lost due to premature death (Table 2), we estimated influenza-associated deaths using mortality data from Statistics Canada's Mortality Database [13]. We considered deaths due to all respiratory and circulatory conditions for seven influenza seasons covering the years from 1997 to 2004. After UIIP introduction, influenza-associated mortality decreased 77% in Ontario compared to 46% in other provinces [13].

The average life expectancy was estimated by age [19] and adjusted for quality of life, using utility scores from a community-dwelling population, ranging from 0.88 to 0.94 depending on age [20].

Quality-adjusted life expectancy was discounted at 3% per year in the base case analysis [21].

### Unit Costs: UIIP Program, Physician Services, and Hospitalizations

All costs were obtained from Ontario sources and are expressed in 2006 Canadian dollars. Cost data are summarized in Table 2.

The total program cost for the universal program was $40 million per year (approximately $3.96 per dose and $7.55 in total per dose distributed) for each year since introducing the program (Nancy Peroff-Johnston, Ministry of Health and Long-Term Care [MOHLTC], personal communication, May 11, 2007). This included the cost of the vaccine, health care provider reimbursement for vaccination, communications strategies, and direct operating expenditures for the MOHLTC including staffing. Approximately 50% of the total budget was spent on the vaccine and the remaining 50% on all other cost items. To calculate the expected targeted program (TIIP) cost if universal immunization (UIIP) had not been implemented, the relative change in vaccine coverage (pre-2000 versus post-2000) observed in other provinces was applied to Ontario's pre-2000 vaccine coverage rate. Vaccine coverage rates for the population 12 y or older more than doubled (18% to 42%) in Ontario and other provinces (13% to 28%) between 1996/1997 and 2005. The Ontario pre-2000 TIIP program cost (Nancy Peroff-Johnston, MOHLTC, personal communication, May 11, 2007) per person vaccinated was used, inflated to 2006, and applied to the population who would have been covered under a targeted program [22]. This assumes that the average cost per person (including vaccine, vaccine delivery, and other cost to the Ministry) remains the same but is applied to a larger number of persons receiving influenza immunization. For an expected TIIP coverage rate of 40%, the total program cost for targeted immunization was expected to be $20 million if TIIP had been continued.

Unit costs for physician services (office visits, ED visits, and in-hospital services) were obtained from the OHIP dataset. A location code in the dataset indicates whether the services were provided in the physician office, ED, or hospital.

Costs reflect the fee paid by condition type (pneumonia and influenza). Mean fees paid ($35 per office visit, $54 per ED visit) were used in the base case analysis; 95% CI defined the lower and upper limit for deterministic sensitivity analysis. For probabilistic analysis the primary data were sampled. An additional $166 per ED visit was added for non-physician costs to calculate total cost per ED visit [23].

Hospitalization costs were costed using the resource intensity weight (RIW) approach [24],[25]. The RIW is the ratio of the cost of a case in a Case Mix Group (CMG) to the average cost of all cases. The mean RIWs for pneumonia- or influenza-related hospitalizations were extracted from the acute care Discharge Abstract Database (DAD). The provincial cost per weighted case of $4,732 was applied to the mean RIW of influenza-associated hospitalizations of 1.33 [23]. Mean physician charges for hospital inpatient services for pneumonia or influenza (obtained from the OHIP database using location code) were added. On average, there were 1.65 claims for physician services per hospitalization at a cost of $66.41 each. The average length of stay for pneumonia and influenza is 6 d [26]. The total mean cost of a pneumonia- and influenza-related episode of hospitalization was therefore $6,418.

### Analyses

#### Cost-effectiveness

Cost-effectiveness of UIIP versus TIIP was measured by the incremental cost-utility ratio (ICUR), defined as the additional cost per QALY gained. The health benefits of universal immunization were estimated as number of influenza cases, health services utilization (physician visits, ED visits, hospitalizations), and deaths prevented in a typical influenza season. A time horizon of a lifetime was adopted to calculate QALYs lost attributable to influenza-related death. Time preference was incorporated by discounting QALYs lost at 3% annually in the base case analysis [21]. Costs are not discounted because all costs occur within 1 y. The analysis was performed from the perspective of the health care payer, the Ontario MOHLTC.

#### Sensitivity analysis

The impact of data uncertainty was explored with one-way and probabilistic sensitivity analyses. One-way sensitivity analyses were performed for numbers of events, number of cases, event costs, immunization program cost, QALY penalty associated with influenza, and discount rate (undiscounted and 5%). Grouped sensitivity analysis was performed by simultaneously adjusting the values of (1) events (all events and deaths separate), (2) relative rate (RR) of events (office visits, ED visits, hospitalizations, and deaths, each event separate), (3) costs of events (office visits, ED visits, and hospitalizations, each event separate), and (4) QALY gain due to morbidity (illness) averted. This was achieved by the use of scaling parameters that link variables within a group and allow the simultaneous adjustment of their values over a defined range. The base case value was therefore multiplied with scaling parameter values ranging from 0 to 2. This range covers the 95% CI of all parameters except for deaths observed post-UIIP in persons under 65 y of age. The range for post-UIIP deaths is −4 to 6 for <65 y old. This age group (<65 y) accounts for approximately 2% of all deaths (7 out of 309 deaths per season). We therefore assumed the range from 0 to 2 for scaling parameters to be acceptable for varying post-UIIP deaths for all age groups combined (not individually). Two extreme (best and worst) case scenarios were also performed (Tables 1 and 2; more detailed information can be found in Tables S1 and S2) biasing the analysis for and against UIIP. The 95% CIs were used as extreme values for pre- and post-UIIP number of events (cases, physician services, ED visits, hospitalizations, deaths), RRs of events (to calculate number of events if TIIP would have been continued), unit costs, and QALY loss attributable to influenza morbidity.

For probabilistic analysis, distributions were assigned to key variables in the model (Tables S1 and S2). Distributions for events (office visits, ED visits, hospitalizations, and deaths), RRs, and unit cost for events were drawn from the original data. For unit costs for physician services, the actual administrative data were sampled using $10.00 increments, with the last bin being $220 per visit and greater (fees paid: $0 to <$10, $10 to <$20, $20 to <$30, … , $210 to <$220, $220 and greater). The midpoint for the top and bottom categories was adjusted by the minimum and maximum fee charged. The midpoint for the bottom category was $5.50 for office visits, $9.00 for ED visits, and $6.50 for inpatient services. The midpoint for the top category was $225 for office visits and inpatient services and $222 for ED visits. The midpoint of each bin was used for calculation. For hospitalization cost, the RIW 95% CI defined the lower and upper limit for deterministic sensitivity analysis. For probabilistic analysis, the primary data were sampled. Distributions for utility weights were based on confidence intervals in published reports [16]. The probability of Ontario's UIIP being cost-effective for a range of willingness to pay thresholds ($0 to $100,000 per QALY gained) was calculated by sampling from these distributions running 1,000 trials. To ensure internal consistency, the same random number was used to sample from distributions for the different age brackets within the same group of variables: pre-UIIP events, post-UIIP events, RR, and QALY gain due to morbidity averted.

An additional analysis, using the diagnostic codes for all respiratory conditions to extract health care utilization data, is presented in the Text S2. This analysis was performed because many influenza-related health care interactions are coded under different diagnostic codes (e.g., common cold, asthma, COPD, otitis media, etc.), therefore underestimating the health care resource use due to influenza if using the diagnostic codes for pneumonia and influenza only (as in all other analyses).

## Results

### Base Case

In Ontario, 22,457 cases of influenza were observed on average per season after the introduction of the universal immunization program. If TIIP had been continued, the expected average number of cases per season was estimated to be 56,998. UIIP therefore prevented 34,541 influenza cases (61% of all cases each season).

Ontario's UIIP also prevented 111 deaths, a 28% reduction in mortality. This resulted in a projected 1,134 QALYs gained in total or 0.09 quality-adjusted life days per person vaccinated. Approximately half of all health gains (QALYs) were associated with a reduction of influenza mortality; the other half was associated with reduction in influenza-related morbidity.

The program costs of UIIP are high, approximately double that of a targeted program ($40 million versus $20 million). However, UIIP was estimated to prevent 786 influenza-related hospitalizations, 7,745 influenza-related ED visits, and 30,306 office visits per season. Preventing influenza cases effectively reduced influenza-related health care costs by 52%, saving the health care system approximately $7.8 million per season, so that the net cost of the UIIP program is $12.2 million, or $2.60 per person vaccinated.

The cost per QALY gained is $10,797/QALY (discount rate 3%). All base case results are presented in Table 3 and in more detail in Table S3.

**Table 3:** Results—base case.

Outcome Measure	TIIP	UIIP	Incremental (UIIP–TIIP)	ICER ($/QALY)
Immunization program cost ($M)	$19.95	$40.00	$20.05	
**Cost ($M)**				
Office visits	$1.75	$0.68	−$1.07	
ED visits	$2.78	$1.07	−$1.70	
Hospitalizations	$10.87	$5.83	−$5.04	
Total	$15.40	$7.58	−$7.81	
Net cost ($M)			$12.24	
**Resource use**				
Office visits	49,638	19,332	−30,306	
ED visits	12.627	4,882	−7,745	
Hospitalizations	1,694	908	−786	
**Health outcome**				
Cases	56,931	22,390	−34,541	
Deaths	394	283	−111	
**QALYs (undiscounted)**				
Morbidity	−964	−412	552	
Mortality	−2,324	−1,291	1,033	
Total	−3,289	−1,703	1,585	$7,721
**QALYs (discounted 3%)**				
Morbidity	−964	−412	552	
Mortality	−1,576	−994	581	
Total	−2,540	−1,406	1,134	$10,797
**QALYs (discounted 5%)**				
Morbidity	−964	−412	552	
Mortality	−1,330	−875	455	
Total	−2,294	−1,287	1,007	$12,154

ED, emergency department; ICER, incremental cost-effectiveness ratio; $M, $ million; QALY, quality-adjusted life years; TIIP, targeted influenza immunization program; UIIP, universal influenza immunization program.

### Sensitivity Analysis

**Figure 1:**
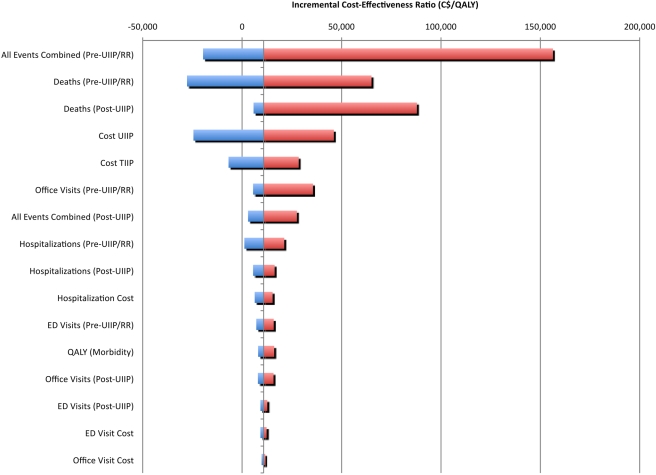
Tornado diagram comparing the relative importance of variables. The grey vertical line corresponds to all the uncertain parameters being at their respective base values. The width of the bars indicates the uncertainty associated with each parameter (ranging from lower to upper limit). The red segments of the bars correspond to result values increasing the base case ICER, and the blue segments of the bars correspond to result values decreasing the base case ICER. ED, emergency department; QALY, quality-adjusted life year; RR, relative rate; TIIP, targeted influenza immunization program; UIIP, universal influenza immunization program.

Deterministic one-way sensitivity analysis (Figure 1, Table S4) revealed that results are highly sensitive to the RR of deaths if TIIP had been continued, the pre-UIIP and post-UIIP number of deaths, and moderately sensitive to UIIP and TIIP immunization program cost. However, the ICER remains below $50,000 per QALY under the following conservative individual conditions: (1) UIIP costs are twice the current program cost, (2) TIIP costs are zero, (3) the RR of death is not smaller than 45% of the base case value, (4) the pre-UIIP number of deaths is not smaller than 45% of the base case value, or (5) the post-UIIP number of deaths is not greater than 190% of the base case value.

It is highly unlikely that these thresholds for key parameters are exceeded. Program costs were obtained from the Ontario Ministry of Health and thus reflect the true costs to the Ministry. A RR of death or a pre-UIIP number of deaths smaller than 45% of the base case value is far outside the 95% CI for these values. The 95% CIs range from 92% to 108% of the base case value for pre-UIIP deaths (all ages) and from 85% to 118% of base case value for RR of death (all ages). Similarly a post-UIIP number of deaths greater than 190% of the base case value is outside the 95% CI range for this variable (−260% to 120% of the base case value).

An analysis of extremes was also performed by examining best- and worst-case scenarios (Table S5). In the worst-case scenario (UIIP does not provide much health benefit over TIIP, health service resource unit costs are low), UIIP increases program costs by $20 million, saves only $1.8 million in health care service cost, and incurs a QALY loss. UIIP becomes unattractive because it is not associated with health gain, and also increased costs compared to TIIP.

In the best-case scenario (UIIP is highly effective, health service resource unit costs are high), UIIP increases program costs by $19.1 million, saves $40.4 million in health care services, and gains 5,619 QALYs (discount rate 3%). UIIP dominates TIIP in this scenario; i.e., UIIP is less costly and more effective than TIIP.

**Figure 2:**
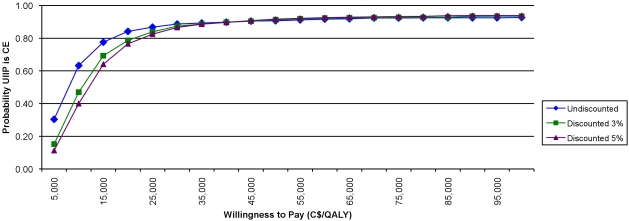
Incremental cost acceptability curve.

Probabilistic sensitivity analysis (Table 4, Figure 2) shows that the probability of UIIP being cost-effective at a willingness-to-pay threshold of $50,000 per QALY is exceeding 90%. The mean (95% CI) QALYs gained are 1,263 [−456; 3,082], and the mean (95% CI) net cost is $12.10 million [−$0.01 million; $16.11 million]. Finally, results for the additional analysis, using the diagnostic codes for all respiratory conditions to extract health care utilization data, are presented in Table S7.

**Table 4:** Results—probabilistic sensitivity analysis.

Analysis	Net Cost ($M)	Net QALYs^a^
Deterministic	$12.24	1,134
Probabilistic		
Mean	$12.10	1,263
Median	$13.46	1,190
2.5th percentile	−$0.01	−456
97.5th percentile	$16.11	3,082

a Discounted at an annual rate of 3%.

$M, $ million; QALY, quality-adjusted life years.

## Discussion

Our analysis suggests that Ontario's UIIP is economically attractive. UIIP reduces the number of influenza cases and deaths and reduces health services resource use. The additional costs of UIIP are partly (39%) offset by savings in health care costs. Compared to TIIP, UIIP is cost-effective at an ICUR of $10,797 per QALY gained.

The main limitation in determining the impact of UIIP is the ecologic study design of the underlying effectiveness study. Causality cannot be established with this type of study design. The effect size may be exaggerated if there are province-specific epidemiological or health service factors that have in more recent years reduced influenza events in Ontario or increased it in other provinces. Similarly, since the outcome measure is influenza-like illness rather than confirmed influenza, it is possible that this could make estimates of outcomes sensitive to changes in the epidemiology of non-influenza respiratory illness. However, this design is appropriate for assessing the public health impact of a population-wide intervention [13]. The results of Kwong's analysis of observational ecologic data in four provinces over 7 y are congruent with the results of randomized controlled trial data in targeted groups. Targeted immunization has been shown to be effective in preventing laboratory-confirmed influenza infection in healthy children 2 y and older [27], in healthy adults, especially when there is a good match and virus circulation is high [28], and older adults (65 y and older), especially in long-term care facilities [28]. The weight of evidence seems to support the hypothesis that UIIP has been responsible for the decrease in cases and deaths in Ontario, in the absence of other obvious causal mechanisms. Stronger randomised clinical trial evidence of the effectiveness of universal vaccination in large populations is unlikely to be available in the future. Finally, to test whether the disproportionate decrease in influenza events from a 9% difference in coverage improvement may be attributable to herd immunity, we analyzed a simple Susceptible-Infected-Removed (SIR) compartmental model (for a description of the model and results, see Figures S1 and S2, Tables S7 and S8, and Text S3). This basic analysis suggests that the 40%–60% decrease in influenza events is plausible.

Other study limitations relate to the definition of cases and quality of life estimates used. Cases were defined based on health care resource use for pneumonia and influenza and therefore represent influenza-like illness cases. Symptomatic cases not requiring health care contacts are not included, potentially underestimating the benefit of the program. Utility weights to estimate QALYs were obtained from an analysis of clinical trial data from influenza patients in Europe and North America [16]. While these data are not Canada-specific, the populations are similar. Furthermore, sensitivity analysis was performed and reported for utility weights used in the analysis.

This study has several strengths. The model is robust and is based on administrative health care resource use and cost data covering all residents of Ontario. Data were extracted and analyzed in detail to estimate influenza-associated events and costs as accurately as possible to assess the impact of this intervention on the population of interest rather than estimating parameter values from other, smaller subpopulations that may not be representative. Finally, extensive sensitivity analyses demonstrated the program to be effective and cost-effective under very conservative assumptions.

The cost-effectiveness of influenza immunization programs has been demonstrated by numerous economic evaluations of TIIPs, many of which are directly based on clinical trial data. TIIPs have been shown to be cost-effective in children 6 mo and older [29]–[36], adults 50 y and older [37]–[41], working adults [42]–[44], working adult cancer patients [45], pregnant women [46], health care workers [47],[48], high-risk individuals [49], and older adults (65 y and older) [39],[50]–[55] from a health care payer perspective. Most economic evaluations found TIIPs to be not only cost-effective but cost-saving from a societal perspective.

Policy makers in many jurisdictions considering the implementation of universal immunization have expressed interest in an economic evaluation of Ontario's program. The program appears to offer some health benefits, but the relationship between cost and health benefits of universal immunization had not been evaluated. Our study provides evidence that a universal program is economically attractive in jurisdictions with influenza epidemiology and health care costs that are broadly similar to that of Ontario.

A UIIP may be an appealing intervention in high-income jurisdictions with comparable demographic characteristics (age distribution, risk profile, density) where influenza transmission can be expected to be reasonably similar to the population analyzed. A health care system similar to Ontario's (i.e., health care systems with one major payer), where the costs of the immunization program and the costs of treating influenza cases are both in the payer's budget, will enable the universal program costs to be partly offset by savings in health care cost.

### Conclusion

This analysis indicates that compared to a TIIP, Ontario's UIIP reduces influenza illness attack rates, morbidity, and mortality at reasonable cost to the health care payer.

## Supporting Information

Figure S1 Mean filtered relative reduction in Ontario for a range of vaccine coverage under UIIP.
(0.20 MB TIF)

Figure S2 Proportion of simulations with a relative reduction below 0.01%, 0.1%, 1%, and 5% in Ontario for a range of vaccine coverage under UIIP.
(0.26 MB TIF)

Table S1 Mean annual influenza-related event rates by age group.
(0.14 MB DOC)

Table S2 Relative rates by age groups.
(0.10 MB DOC)

Table S3 Results (base case, disaggregated).
(0.12 MB DOC)

Table S4 Deterministic sensitivity analysis.
(0.29 MB DOC)

Table S5 Aggregated worst-case and best-case results.
(0.08 MB DOC)

Table S6 Results of the regression analysis.
(0.35 MB DOC)

Table S7 Results - sensitivity analysis using all respiratory conditions as outcome.
(0.05 MB DOC)

Table S8 Input parameter ranges.
(0.04 MB DOC)

Table S9 Results of empirical versus predicted relative reduction.
(0.03 MB DOC)

Text S1 Effectiveness of Ontario's UIIP.
(0.06 MB DOC)

Text S2 Additional analysis using all respiratory conditions as outcome.
(0.05 MB DOC)

Text S3 Dynamic transmission model demonstrating herd immunity effects.
(0.07 MB DOC)

## Abbreviations

CMG: Case Mix Group
DAD: Discharge Abstract Database
ED: emergency department
GBS: Guillain-Barré syndrome
ICUR: incremental cost-utility ratio
MOHLTC: Ministry of Health and Long-Term Care
NICE: National Institute for Health and Clinical Excellence
OHIP: Ontario Health Insurance Plan
QALY: quality-adjusted life year
RIW: resource intensity weight
RR: relative rate
SIR: Susceptible-Infected-Removed
TIIP: targeted influenza immunization program
UIIP: universal influenza immunization program
