# Supplementary figures and images for: Economic Appraisal of Ontario's Universal Influenza Immunization Program: A Cost-Utility Analysis
Source: PLoS Med. 2010 Apr 6;7(4):e1000256. doi: 10.1371/journal.pmed.1000256 (PMC2850382; doi:10.1371/journal.pmed.1000256)

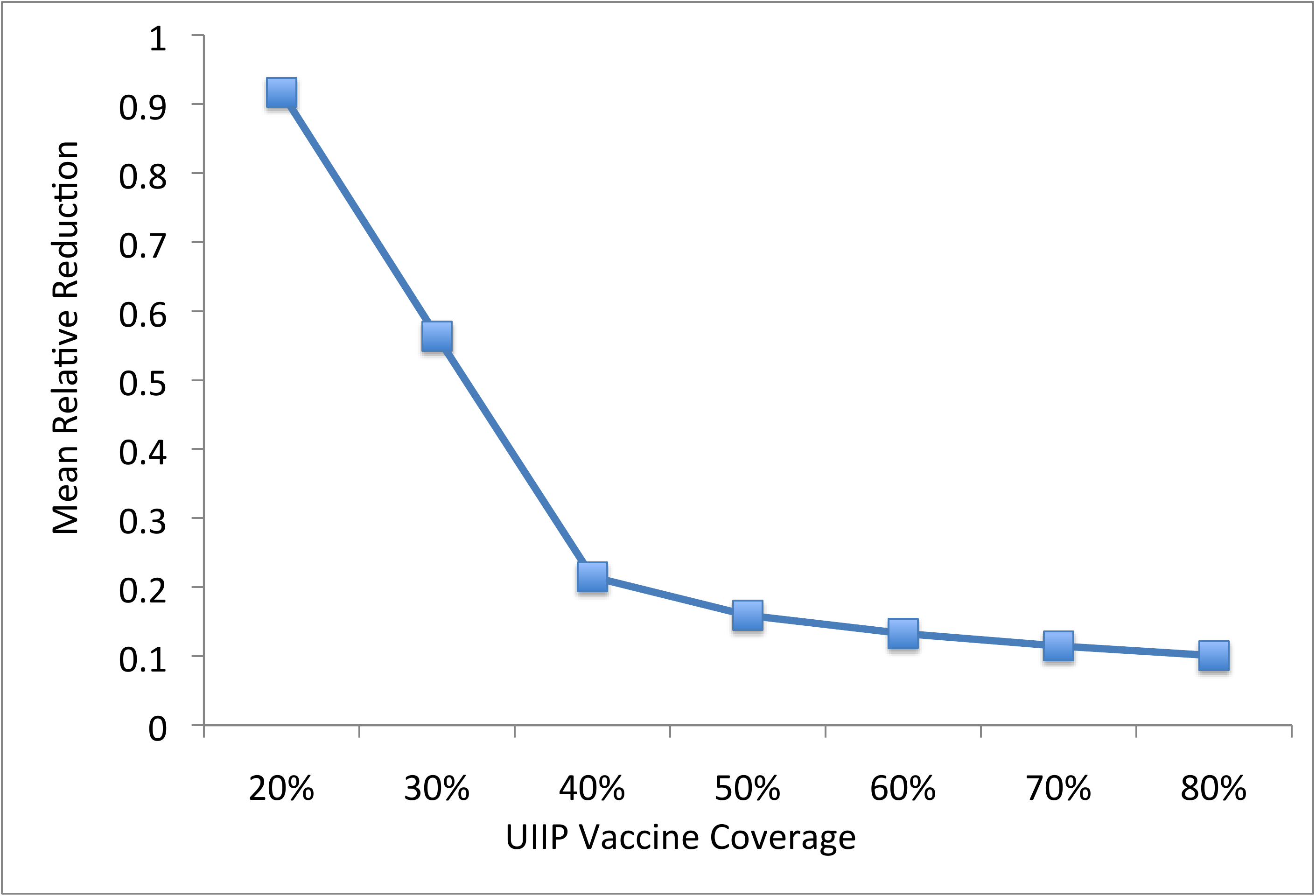

Supplement: Figure S1 — Mean filtered relative reduction in Ontario for a range of vaccine coverage under UIIP. (0.20 MB TIF) [file pmed.1000256.s001.tif]

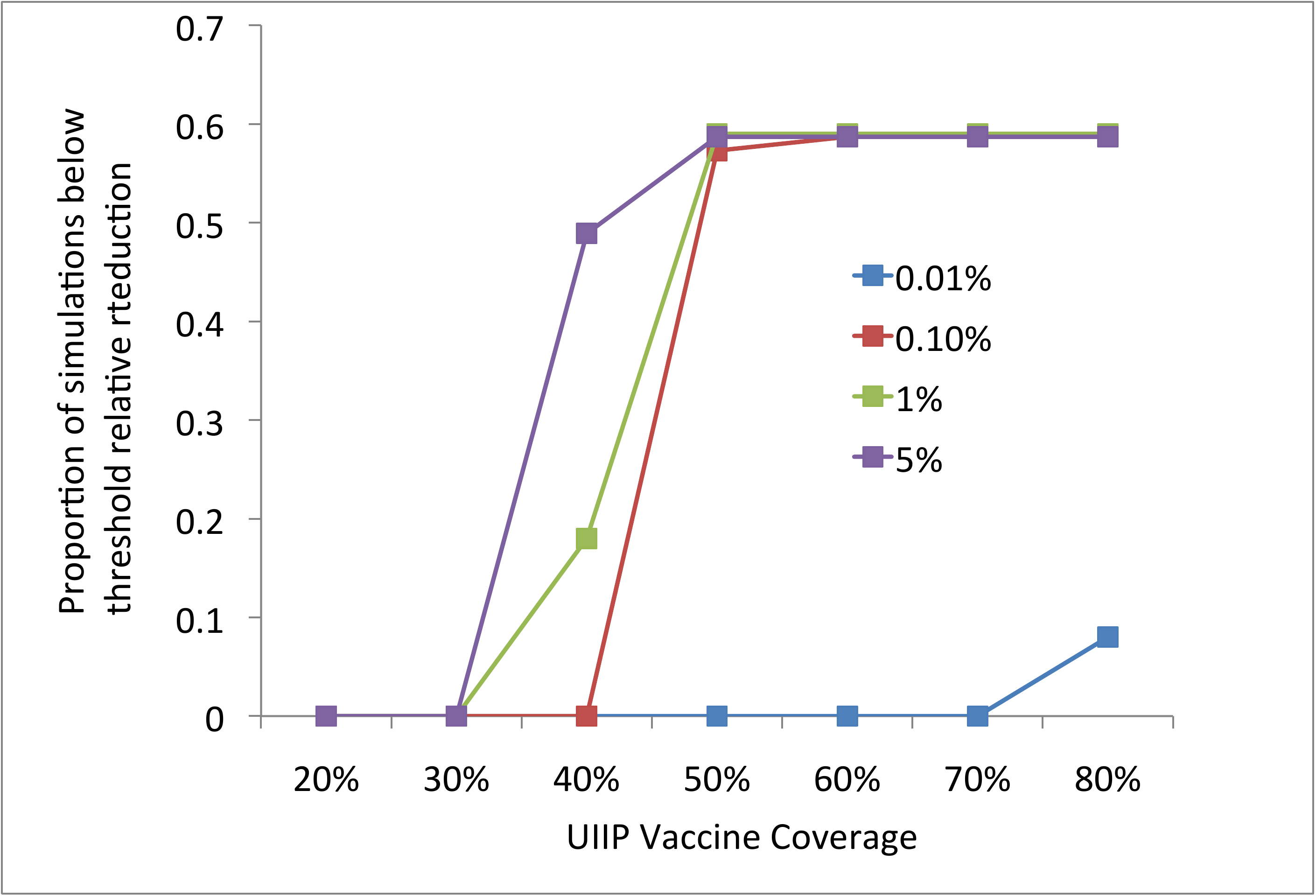

Supplement: Figure S2 — Proportion of simulations with a relative reduction below 0.01%, 0.1%, 1%, and 5% in Ontario for a range of vaccine coverage under UIIP. (0.26 MB TIF) [file pmed.1000256.s002.tif]

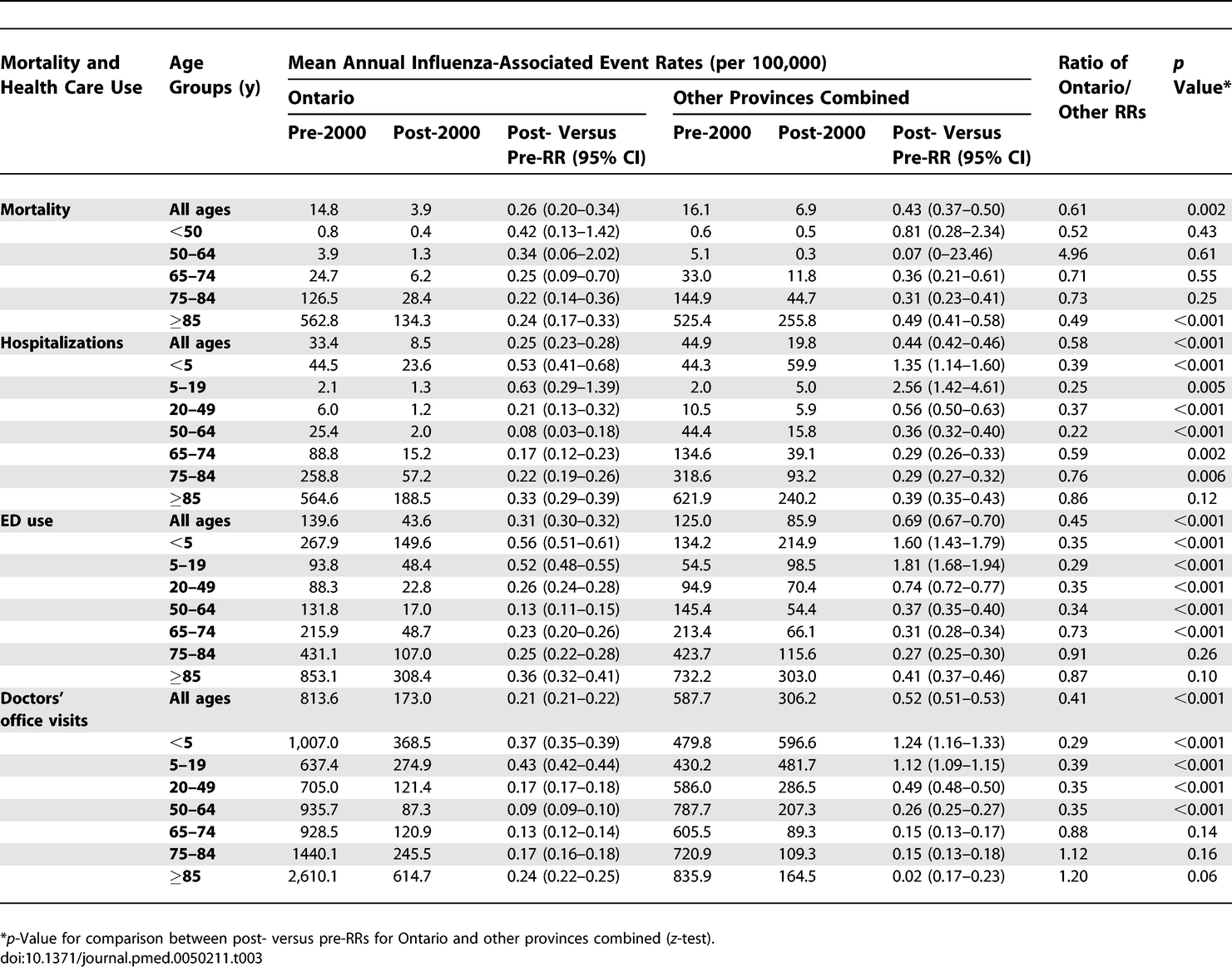

Supplement: Table S6 — Results of the regression analysis. (0.35 MB DOC) [file pmed.1000256.s008.doc]
