# Supplementary material for: Economic Appraisal of Ontario's Universal Influenza Immunization Program: A Cost-Utility Analysis
Source: PLoS Med. 2010 Apr 6;7(4):e1000256. doi: 10.1371/journal.pmed.1000256 (PMC2850382; doi:10.1371/journal.pmed.1000256)
Supplement: Table S1 — Mean annual influenza-related event rates by age group. (0.14 MB DOC) [file pmed.1000256.s003.doc]

| **Table S1:** Mean annual influenza-related event rates by age group | | | | | | | | |
| --- | --- | --- | --- | --- | --- | --- | --- | --- |
|  |  |  | **Base Case Analysis** | **Deterministic Sensitivity Analysis** | | | **Probabilistic Sensitivity Analysis** | |
|  |  |  | **Mean** | | **Lower Confidence Limit**  **(Worst Case)** | **Upper Confidence Limit**  **(Best Case)** | **Distribution** | **Standard Error** |
| Events Pre-UIIP | | | | | | | | |
|  | Office visits | | | | | | | |
|  |  | 0-4 yrs | 7,503 | | 7,289 | 7,718 | normal | 110 |
|  |  | 5-19 yrs | 14,168 | | 13,900 | 14,435 | normal | 137 |
|  |  | 20-49 yrs | 36,494 | | 36,079 | 36,907 | normal | 211 |
|  |  | 50-64 yrs | 14,902 | | 14,682 | 15,123 | normal | 113 |
|  |  | 65-74 yrs | 7,436 | | 7,273 | 7,599 | normal | 83 |
|  |  | 75-84 yrs | 6,128 | | 5,988 | 6,268 | normal | 71 |
|  |  | 85+ yrs | 3,348 | | 3,249 | 3,447 | normal | 50 |
|  | ED visits | | | | | | | |
|  |  | 0-4 yrs | 1,996 | | 1,894 | 2,099 | normal | 52 |
|  |  | 5-19 yrs | 2,086 | | 2,003 | 2,170 | normal | 43 |
|  |  | 20-49 yrs | 4,571 | | 4,452 | 4,690 | normal | 61 |
|  |  | 50-64 yrs | 2,099 | | 2,023 | 2,176 | normal | 39 |
|  |  | 65-74 yrs | 1,729 | | 1,651 | 1,806 | normal | 39 |
|  |  | 75-84 yrs | 1,834 | | 1,754 | 1,914 | normal | 41 |
|  |  | 85+ yrs | 1,094 | | 1,033 | 1,155 | normal | 31 |
|  | Hospitalizations | | | | | | | |
|  |  | 0-4 yrs | 331 | | 282 | 381 | normal | 25 |
|  |  | 5-19 yrs | 47 | | 22 | 73 | gamma | 13 |
|  |  | 20-49 yrs | 308 | | 269 | 347 | normal | 20 |
|  |  | 50-64 yrs | 404 | | 360 | 448 | normal | 22 |
|  |  | 65-74 yrs | 711 | | 653 | 770 | normal | 30 |
|  |  | 75-84 yrs | 1,101 | | 1,034 | 1,168 | normal | 34 |
|  |  | 85+ yrs | 724 | | 671 | 777 | normal | 27 |
|  | Deaths | | | | | | | |
|  |  | 0-49 yrs | 20 | | 4 | 37 | gamma | 8 |
|  |  | 50-64 yrs | 67 | | 38 | 96 | normal | 15 |
|  |  | 65-74 yrs | 174 | | 129 | 219 | normal | 23 |
|  |  | 75-84 yrs | 424 | | 365 | 484 | normal | 30 |
|  |  | 85+ yrs | 600 | | 537 | 663 | normal | 32 |
| Events Post-UIIP Observed | | | | | | | | |
|  | Office visits | | | | | | | |
|  |  | 0-4 yrs | 2,746 | | 2,887 | 2,605 | normal | 72 |
|  |  | 5-19 yrs | 6,110 | | 6,260 | 5,959 | normal | 77 |
|  |  | 20-49 yrs | 6,283 | | 6,507 | 6,062 | normal | 114 |
|  |  | 50-64 yrs | 1,391 | | 1,504 | 1,279 | normal | 57 |
|  |  | 65-74 yrs | 968 | | 1,062 | 875 | normal | 48 |
|  |  | 75-84 yrs | 1,045 | | 1,121 | 968 | normal | 39 |
|  |  | 85+ yrs | 788 | | 842 | 735 | normal | 27 |
|  | ED visits | | | | | | | |
|  |  | 0-4 yrs | 1,115 | | 1,193 | 1,036 | normal | 40 |
|  |  | 5-19 yrs | 1,076 | | 1,127 | 1,025 | normal | 26 |
|  |  | 20-49 yrs | 1,180 | | 1,253 | 1,108 | normal | 37 |
|  |  | 50-64 yrs | 270 | | 315 | 226 | normal | 23 |
|  |  | 65-74 yrs | 390 | | 442 | 338 | normal | 27 |
|  |  | 75-84 yrs | 455 | | 508 | 403 | normal | 27 |
|  |  | 85+ yrs | 396 | | 437 | 354 | normal | 21 |
|  | Hospitalizations | | | | | | | |
|  |  | 0-4 yrs | 176 | | 212 | 140 | normal | 18 |
|  |  | 5-19 yrs | 30 | | 47 | 13 | gamma | 9 |
|  |  | 20-49 yrs | 64 | | 93 | 36 | normal | 15 |
|  |  | 50-64 yrs | 32 | | 59 | 5 | gamma | 14 |
|  |  | 65-74 yrs | 121 | | 159 | 84 | normal | 19 |
|  |  | 75-84 yrs | 243 | | 284 | 203 | normal | 21 |
|  |  | 85+ yrs | 242 | | 275 | 209 | normal | 17 |
|  | Deaths | | | | | | | |
|  |  | 0-49 yrs | 3 | | 15 | -8 | gamma | 6 |
|  |  | 50-64 yrs | 4 | | 22 | -14 | gamma | 9 |
|  |  | 65-74 yrs | 45 | | 74 | 15 | gamma | 15 |
|  |  | 75-84 yrs | 93 | | 130 | 56 | normal | 19 |
|  |  | 85+ yrs | 138 | | 176 | 99 | normal | 20 |
| Abbreviations: ED, emergency department; UIIP, universal influenza immunization program  Note: Table S1 shows the mean annual influenza-related event rates used in the base case and deterministic and probabilistic sensitivity analyses. The rates are observed rates in Ontario before and after introduction of the universal influenza immunization program.  Source: Kwong JC, Stukel TA, Lim J, McGeer AJ, Upshur RE, et al. (2008) The effect of universal influenza immunization on mortality and health care use. PLoS Med 5: e211. | | | | | | | | |
