# Supplementary material for: Economic Appraisal of Ontario's Universal Influenza Immunization Program: A Cost-Utility Analysis
Source: PLoS Med. 2010 Apr 6;7(4):e1000256. doi: 10.1371/journal.pmed.1000256 (PMC2850382; doi:10.1371/journal.pmed.1000256)
Supplement: Table S2 — Relative rates by age groups. (0.10 MB DOC) [file pmed.1000256.s004.doc]

| **Table S2:** Relative rates by Age Groups | | | | | |
| --- | --- | --- | --- | --- | --- |
|  |  | **Base Case Analysis** | **Deterministic Sensitivity Analysis** | | **Probabilistic Sensitivity Analysis** |
|  |  |  | **Lower confidence limit**  **(Worst Case)** | **Upper confidence limit**  **(Best Case)** | **Distribution** |
| Office visits | | | | | |
|  | 0-4 yrs | 1.24 | 1.16 | 1.33 | lognormal |
|  | 5-19 yrs | 1.12 | 1.09 | 1.15 | lognormal |
|  | 20-49 yrs | 0.49 | 0.48 | 0.50 | lognormal |
|  | 50-64 yrs | 0.26 | 0.25 | 0.27 | lognormal |
|  | 65-74 yrs | 0.15 | 0.13 | 0.17 | lognormal |
|  | 75-84 yrs | 0.15 | 0.13 | 0.18 | lognormal |
|  | 85+ yrs | 0.20 | 0.17 | 0.23 | lognormal |
| ED visits | | | | | |
|  | 0-4 yrs | 1.60 | 1.43 | 1.79 | lognormal |
|  | 5-19 yrs | 1.81 | 1.68 | 1.94 | lognormal |
|  | 20-49 yrs | 0.74 | 0.72 | 0.77 | lognormal |
|  | 50-64 yrs | 0.37 | 0.35 | 0.40 | lognormal |
|  | 65-74 yrs | 0.31 | 0.28 | 0.34 | lognormal |
|  | 75-84 yrs | 0.27 | 0.25 | 0.30 | lognormal |
|  | 85+ yrs | 0.41 | 0.37 | 0.46 | lognormal |
| Hospitalizations | | | | | |
|  | 0-4 yrs | 1.35 | 1.14 | 1.60 | lognormal |
|  | 5-19 yrs | 2.56 | 1.42 | 4.61 | lognormal |
|  | 20-49 yrs | 0.56 | 0.50 | 0.63 | lognormal |
|  | 50-64 yrs | 0.36 | 0.32 | 0.40 | lognormal |
|  | 65-74 yrs | 0.29 | 0.26 | 0.33 | lognormal |
|  | 75-84 yrs | 0.29 | 0.27 | 0.32 | lognormal |
|  | 85+ yrs | 0.39 | 0.35 | 0.43 | lognormal |
| Deaths | | | | | |
|  | 0-49 yrs | 0.67 | 0.24 | 1.90 | lognormal |
|  | 50-64 yrs | 0.16 | 0.03 | 0.84 | lognormal |
|  | 65-74 yrs | 0.32 | 0.21 | 0.48 | lognormal |
|  | 75-84 yrs | 0.26 | 0.19 | 0.35 | lognormal |
|  | 85+ yrs | 0.34 | 0.28 | 0.43 | lognormal |
| Abbreviations: ED, emergency department; RR, relative rate  Note: Relative rates were used to calculate the expected mean annual number of events in Ontario had Ontario continued to offer a targeted influenza immunization program instead of a universal program. The relative rate estimates were calculated from observed number of events before and after 2000 in other provinces that continued to offer targeted influenza immunization programs.  Source: Kwong JC, Stukel TA, Lim J, McGeer AJ, Upshur RE, et al. (2008) The effect of universal influenza immunization on mortality and health care use. PLoS Med 5: e211. | | | | | |
