# Supplementary material for: Economic Appraisal of Ontario's Universal Influenza Immunization Program: A Cost-Utility Analysis
Source: PLoS Med. 2010 Apr 6;7(4):e1000256. doi: 10.1371/journal.pmed.1000256 (PMC2850382; doi:10.1371/journal.pmed.1000256)
Supplement: Table S3 — Results (base case, disaggregated). (0.12 MB DOC) [file pmed.1000256.s005.doc]

| **Table S3:** Results (base case, disaggregated) | | | | | | |
| --- | --- | --- | --- | --- | --- | --- |
|  |  |  | **Events Prevented** | **Cost Avoided** | **Cases Prevented** | **QALYs gained** |
| Total | | | | | | |
|  | Undiscounted | |  | $7,812,139 | 34,541 | 1,585 |
|  | Discounted 3% | |  |  |  | 1,134 |
|  | Discounted 5% | |  |  |  | 1,007 |
| Office visits | | | | | | |
|  | Total | | 30,306 | 1,066,390 | 27,141 | 431 |
|  | 0-4 yrs | | 6,585 | $231,705 |  | 86 |
|  | 5-19 yrs | | 9,752 | $343,143 |  | 128 |
|  | 20-49 yrs | | 11,555 | $406,602 |  | 180 |
|  | 50-64 yrs | | 2,531 | $89,054 |  | 39 |
|  | 65-74 yrs | | 128 | $4,490 |  | 3 |
|  | 75-84 yrs | | -115 | -$4,045 |  | -3 |
|  | 85+ yrs | | -130 | -$4,559 |  | -3 |
| ED visits | | | | | | |
|  | Total | | 7,745 | 1,703,387 | 6,614 | 106 |
|  | 0-4 yrs | | 2,081 | $457,742 |  | 26 |
|  | 5-19 yrs | | 2,690 | $591,625 |  | 34 |
|  | 20-49 yrs | | 2,211 | $486,244 |  | 33 |
|  | 50-64 yrs | | 515 | $113,325 |  | 8 |
|  | 65-74 yrs | | 145 | $31,935 |  | 4 |
|  | 75-84 yrs | | 45 | $9,931 |  | 1 |
|  | 85+ yrs | | 57 | $12,586 |  | 1 |
| Hospitalizations | | | | | | |
|  | Total | | 786 | 5,042,362 | 786 | 15 |
|  | 0-4 yrs | | 272 | $1,744,156 |  | 4 |
|  | 5-19 yrs | | 91 | $583,278 |  | 1 |
|  | 20-49 yrs | | 109 | $699,794 |  | 2 |
|  | 50-64 yrs | | 112 | $718,968 |  | 2 |
|  | 65-74 yrs | | 85 | $547,766 |  | 3 |
|  | 75-84 yrs | | 79 | $505,575 |  | 2 |
|  | 85+ yrs | | 38 | $242,826 |  | 1 |
| Deaths | | | | | | |
|  | Undiscounted | | | | | |
|  |  | Total | 111 |  |  | 1,033 |
|  |  | 0-49 yrs | 11 |  |  | 657 |
|  |  | 50-64 yrs | 6 |  |  | 135 |
|  |  | 65-74 yrs | 11 |  |  | 111 |
|  |  | 75-84 yrs | 15 |  |  | 68 |
|  |  | 85+ yrs | 68 |  |  | 62 |
|  | Discounted 3% | | | | | |
|  |  | Total | 111 |  |  | 581 |
|  |  | 0-49 yrs | 11 |  |  | 283 |
|  |  | 50-64 yrs | 6 |  |  | 93 |
|  |  | 65-74 yrs | 11 |  |  | 88 |
|  |  | 75-84 yrs | 15 |  |  | 59 |
|  |  | 85+ yrs | 68 |  |  | 59 |
|  | Discounted 5% | | | | | |
|  |  | Total | 111 |  |  | 455 |
|  |  | 0-49 yrs | 11 |  |  | 192 |
|  |  | 50-64 yrs | 6 |  |  | 75 |
|  |  | 65-74 yrs | 11 |  |  | 76 |
|  |  | 75-84 yrs | 15 |  |  | 55 |
|  |  | 85+ yrs | 68 |  |  | 57 |
| Abbreviations: ED, emergency department; QALY, quality adjusted life year  Note: This table shows the base case results by age groups: ≤4 years, 5 to 19 years, 20 to 49 years, 50 to 64 years, 65 to 74 years, 75 to 84 years and 85 years or older. | | | | | | |
