# Supplementary material for: Economic Appraisal of Ontario's Universal Influenza Immunization Program: A Cost-Utility Analysis
Source: PLoS Med. 2010 Apr 6;7(4):e1000256. doi: 10.1371/journal.pmed.1000256 (PMC2850382; doi:10.1371/journal.pmed.1000256)
Supplement: Table S4 — Deterministic sensitivity analysis. (0.29 MB DOC) [file pmed.1000256.s006.doc]

| **Table S4:** Deterministic Sensitivity Analysis | | | | |
| --- | --- | --- | --- | --- |
|  | **Scaling Factor** | **Net QALYs** | **Net Cost** | **ICER (Cost/QALY)** |
| Baseline | | | | |
|  | 1.0 | 1,134 | $12,241,305 | $10,797 |
| Events Pre-UIIP: All | | | | |
|  | 0.0 | 170 | $27,637,357 | $163,003 |
|  | 0.2 | 362 | $24,558,147 | $67,766 |
|  | 0.4 | 555 | $21,478,936 | $38,684 |
|  | 0.6 | 748 | $18,399,726 | $24,596 |
|  | 0.8 | 941 | $15,320,515 | $16,282 |
|  | 1.0 | 1,134 | $12,241,305 | $10,797 |
|  | 1.2 | 1,327 | $9,162,094 | $6,906 |
|  | 1.4 | 1,519 | $6,082,884 | $4,003 |
|  | 1.6 | 1,712 | $3,003,673 | $1,754 |
|  | 1.8 | 1,905 | -$75,537 | UIIP Dominant |
|  | 2.0 | 2,098 | -$3,154,748 | UIIP Dominant |
| Events Pre-UIIP: Death | | | | |
|  | 0.0 | -442 | $12,241,305 | TIIP Dominant |
|  | 0.2 | -127 | $12,241,305 | TIIP Dominant |
|  | 0.4 | 188 | $12,241,305 | $65,032 |
|  | 0.6 | 503 | $12,241,305 | $24,317 |
|  | 0.8 | 819 | $12,241,305 | $14,954 |
|  | 1.0 | 1,134 | $12,241,305 | $10,797 |
|  | 1.2 | 1,449 | $12,241,305 | $8,448 |
|  | 1.4 | 1,764 | $12,241,305 | $6,939 |
|  | 1.6 | 2,079 | $12,241,305 | $5,887 |
|  | 1.8 | 2,394 | $12,241,305 | $5,112 |
|  | 2.0 | 2,710 | $12,241,305 | $4,518 |
| Events Post-UIIP: All | | | | |
|  | 0.0 | 1,546 | $4,657,391 | $3,013 |
|  | 0.2 | 1,463 | $6,174,174 | $4,219 |
|  | 0.4 | 1,381 | $7,690,957 | $5,569 |
|  | 0.6 | 1,299 | $9,207,739 | $7,091 |
|  | 0.8 | 1,216 | $10,724,522 | $8,818 |
|  | 1.0 | 1,134 | $12,241,305 | $10,797 |
|  | 1.2 | 1,051 | $13,758,087 | $13,086 |
|  | 1.4 | 969 | $15,274,870 | $15,764 |
|  | 1.6 | 887 | $16,791,653 | $18,939 |
|  | 1.8 | 804 | $18,308,435 | $22,766 |
|  | 2.0 | 722 | $19,825,218 | $27,466 |
| Events Post-UIIP: Death | | | | |
|  | 0.0 | 2,128 | $12,241,305 | $5,752 |
|  | 0.2 | 1,929 | $12,241,305 | $6,345 |
|  | 0.4 | 1,730 | $12,241,305 | $7,074 |
|  | 0.6 | 1,532 | $12,241,305 | $7,993 |
|  | 0.8 | 1,333 | $12,241,305 | $9,186 |
|  | 1.0 | 1,134 | $12,241,305 | $10,797 |
|  | 1.2 | 935 | $12,241,305 | $13,094 |
|  | 1.4 | 736 | $12,241,305 | $16,632 |
|  | 1.6 | 537 | $12,241,305 | $22,790 |
|  | 1.8 | 338 | $12,241,305 | $36,189 |
|  | 2.0 | 139 | $12,241,305 | $87,828 |
| RR: All | | | | |
|  | 0.0 | -1,406 | $27,637,357 | TIIP Dominant |
|  | 0.2 | -898 | $24,558,147 | TIIP Dominant |
|  | 0.4 | -390 | $21,478,936 | TIIP Dominant |
|  | 0.6 | 118 | $18,399,726 | $156,292 |
|  | 0.8 | 626 | $15,320,515 | $24,484 |
|  | 1.0 | 1,134 | $12,241,305 | $10,797 |
|  | 1.2 | 1,642 | $9,162,094 | $5,581 |
|  | 1.4 | 2,150 | $6,082,884 | $2,830 |
|  | 1.6 | 2,658 | $3,003,673 | $1,130 |
|  | 1.8 | 3,166 | -$75,537 | UIIP Dominant |
|  | 2.0 | 3,674 | -$3,154,748 | UIIP Dominant |
| RR: Office Visits | | | | |
|  | 0.0 | 394 | $13,987,916 | $35,540 |
|  | 0.2 | 542 | $13,638,594 | $25,181 |
|  | 0.4 | 690 | $13,289,272 | $19,269 |
|  | 0.6 | 838 | $12,939,949 | $15,447 |
|  | 0.8 | 986 | $12,590,627 | $12,773 |
|  | 1.0 | 1,134 | $12,241,305 | $10,797 |
|  | 1.2 | 1,282 | $11,891,982 | $9,278 |
|  | 1.4 | 1,430 | $11,542,660 | $8,073 |
|  | 1.6 | 1,578 | $11,193,338 | $7,094 |
|  | 1.8 | 1,726 | $10,844,015 | $6,283 |
|  | 2.0 | 1,874 | $10,494,693 | $5,600 |
| RR: ED Visits | | | | |
|  | 0.0 | 947 | $15,018,492 | $15,854 |
|  | 0.2 | 985 | $14,463,054 | $14,689 |
|  | 0.4 | 1,022 | $13,907,617 | $13,610 |
|  | 0.6 | 1,059 | $13,352,179 | $12,606 |
|  | 0.8 | 1,096 | $12,796,742 | $11,671 |
|  | 1.0 | 1,134 | $12,241,305 | $10,797 |
|  | 1.2 | 1,171 | $11,685,867 | $9,979 |
|  | 1.4 | 1,208 | $11,130,430 | $9,211 |
|  | 1.6 | 1,246 | $10,574,992 | $8,490 |
|  | 1.8 | 1,283 | $10,019,555 | $7,810 |
|  | 2.0 | 1,320 | $9,464,118 | $7,169 |
| RR: Hospitalizations | | | | |
|  | 0.0 | 1,096 | $23,113,559 | $21,085 |
|  | 0.2 | 1,104 | $20,939,108 | $18,972 |
|  | 0.4 | 1,111 | $18,764,657 | $16,886 |
|  | 0.6 | 1,119 | $16,590,206 | $14,829 |
|  | 0.8 | 1,126 | $14,415,755 | $12,800 |
|  | 1.0 | 1,134 | $12,241,305 | $10,797 |
|  | 1.2 | 1,141 | $10,066,854 | $8,821 |
|  | 1.4 | 1,149 | $7,892,403 | $6,870 |
|  | 1.6 | 1,156 | $5,717,952 | $4,945 |
|  | 1.8 | 1,164 | $3,543,502 | $3,045 |
|  | 2.0 | 1,171 | $1,369,051 | $1,169 |
| RR: Death | | | | |
|  | 0.0 | -442 | $12,241,305 | TIIP Dominant |
|  | 0.2 | -127 | $12,241,305 | TIIP Dominant |
|  | 0.4 | 188 | $12,241,305 | $65,032 |
|  | 0.6 | 503 | $12,241,305 | $24,317 |
|  | 0.8 | 819 | $12,241,305 | $14,954 |
|  | 1.0 | 1,134 | $12,241,305 | $10,797 |
|  | 1.2 | 1,449 | $12,241,305 | $8,448 |
|  | 1.4 | 1,764 | $12,241,305 | $6,939 |
|  | 1.6 | 2,079 | $12,241,305 | $5,887 |
|  | 1.8 | 2,394 | $12,241,305 | $5,112 |
|  | 2.0 | 2,710 | $12,241,305 | $4,518 |
| QALY (Morbidity) | | | | |
|  | 0.0 | 994 | $12,241,305 | $16,069 |
|  | 0.2 | 836 | $12,241,305 | $14,639 |
|  | 0.4 | 911 | $12,241,305 | $13,443 |
|  | 0.6 | 985 | $12,241,305 | $12,428 |
|  | 0.8 | 1,059 | $12,241,305 | $11,555 |
|  | 1.0 | 1,134 | $12,241,305 | $10,797 |
|  | 1.2 | 1,208 | $12,241,305 | $10,132 |
|  | 1.4 | 1,283 | $12,241,305 | $9,545 |
|  | 1.6 | 1,357 | $12,241,305 | $9,021 |
|  | 1.8 | 1,431 | $12,241,305 | $8,552 |
|  | 2.0 | 1,506 | $12,241,305 | $8,130 |
| Cost: Office Visits | | | | |
|  | 0.0 | 1,134 | $13,307,695 | $11,738 |
|  | 0.2 | 1,134 | $13,094,417 | $11,549 |
|  | 0.4 | 1,134 | $12,881,139 | $11,361 |
|  | 0.6 | 1,134 | $12,667,861 | $11,173 |
|  | 0.8 | 1,134 | $12,454,583 | $10,985 |
|  | 1.0 | 1,134 | $12,241,305 | $10,797 |
|  | 1.2 | 1,134 | $12,028,027 | $10,609 |
|  | 1.4 | 1,134 | $11,814,749 | $10,421 |
|  | 1.6 | 1,134 | $11,601,471 | $10,233 |
|  | 1.8 | 1,134 | $11,388,193 | $10,045 |
|  | 2.0 | 1,134 | $11,174,914 | $9,856 |
| Cost: ED Visits | | | | |
|  | 0.0 | 1,134 | $13,944,692 | $12,299 |
|  | 0.2 | 1,134 | $13,604,014 | $11,999 |
|  | 0.4 | 1,134 | $13,263,337 | $11,698 |
|  | 0.6 | 1,134 | $12,922,659 | $11,398 |
|  | 0.8 | 1,134 | $12,581,982 | $11,098 |
|  | 1.0 | 1,134 | $12,241,305 | $10,797 |
|  | 1.2 | 1,134 | $11,900,627 | $10,497 |
|  | 1.4 | 1,134 | $11,559,950 | $10,196 |
|  | 1.6 | 1,134 | $11,219,272 | $9,896 |
|  | 1.8 | 1,134 | $10,878,595 | $9,595 |
|  | 2.0 | 1,134 | $10,537,918 | $9,295 |
| Cost: Hospitalizations | | | | |
|  | 0.0 | 1,134 | $17,283,667 | $15,244 |
|  | 0.2 | 1,134 | $16,275,194 | $14,355 |
|  | 0.4 | 1,134 | $15,266,722 | $13,466 |
|  | 0.6 | 1,134 | $14,258,250 | $12,576 |
|  | 0.8 | 1,134 | $13,249,777 | $11,687 |
|  | 1.0 | 1,134 | $12,241,305 | $10,797 |
|  | 1.2 | 1,134 | $11,232,832 | $9,908 |
|  | 1.4 | 1,134 | $10,224,360 | $9,018 |
|  | 1.6 | 1,134 | $9,215,887 | $8,129 |
|  | 1.8 | 1,134 | $8,207,415 | $7,239 |
|  | 2.0 | 1,134 | $7,198,942 | $6,350 |
| Cost: TIIP | | | | |
|  | 0.0 | 1,134 | $17,283,667 | $15,244 |
|  | 0.2 | 1,134 | $16,275,194 | $14,355 |
|  | 0.4 | 1,134 | $15,266,722 | $13,466 |
|  | 0.6 | 1,134 | $14,258,250 | $12,576 |
|  | 0.8 | 1,134 | $13,249,777 | $11,687 |
|  | 1.0 | 1,134 | $12,241,305 | $10,797 |
|  | 1.2 | 1,134 | $11,232,832 | $9,908 |
|  | 1.4 | 1,134 | $10,224,360 | $9,018 |
|  | 1.6 | 1,134 | $9,215,887 | $8,129 |
|  | 1.8 | 1,134 | $8,207,415 | $7,239 |
|  | 2.0 | 1,134 | $7,198,942 | $6,350 |
| Cost: UIIP | | | | |
|  | 0.0 | 1,134 | -$27,758,695 | UIIP Dominant |
|  | 0.2 | 1,134 | -$19,758,695 | UIIP Dominant |
|  | 0.4 | 1,134 | -$11,758,695 | UIIP Dominant |
|  | 0.6 | 1,134 | -$3,758,695 | UIIP Dominant |
|  | 0.8 | 1,134 | $4,241,305 | $3,741 |
|  | 1.0 | 1,134 | $12,241,305 | $10,797 |
|  | 1.2 | 1,134 | $20,241,305 | $17,853 |
|  | 1.4 | 1,134 | $28,241,305 | $24,909 |
|  | 1.6 | 1,134 | $36,241,305 | $31,965 |
|  | 1.8 | 1,134 | $44,241,305 | $39,022 |
|  | 2.0 | 1,134 | $52,241,305 | $46,078 |
| Abbreviations: ED, emergency department; ICER, incremental cost-effectiveness ratio; QALY, quality adjusted life years; RR, relative rate; TIIP, targeted influenza immunization program; UIIP, universal influenza immunization program  Note: An intervention is dominant if the intervention is more effective and less costly then the comparator.  This table shows results for all deterministic sensitivity analysis, varying groups of input variables using scaling factors ranging from zero to two in 0.2 intervals. A scaling factor of 1.0 represents the base case. | | | | |
