# Supplementary material for: Economic Appraisal of Ontario's Universal Influenza Immunization Program: A Cost-Utility Analysis
Source: PLoS Med. 2010 Apr 6;7(4):e1000256. doi: 10.1371/journal.pmed.1000256 (PMC2850382; doi:10.1371/journal.pmed.1000256)
Supplement: Table S5 — Aggregated worst-case and best-case results. (0.08 MB DOC) [file pmed.1000256.s007.doc]

| **Table S5:** Aggregated Worst Case and Best Case Results | | | | | |
| --- | --- | --- | --- | --- | --- |
|  |  | **Worst Case** | | **Best Case** | |
|  |  | **Incremental**  **(UIIP – TIIP)** | **ICER ($/QALY)** | **Incremental**  **(UIIP – TIIP)** | **ICER ($/QALY)** |
| Immunization Program Cost  ($M) | | $19.33 | $20.67 | $19.06 |  |
| Cost Avoided ($M) | | | | | |
|  | Office Visits | $0.47 |  | $2.03 |  |
|  | ED Visits | $1.09 |  | $3.60 |  |
|  | Hospitalizations | $0.32 |  | $34.81 |  |
|  | Total | $1.88 |  | $40.44 |  |
| Net Cost ($M) | | $18.78 |  | -$21.38 |  |
| Resource Use Prevented | |  |  |  |  |
|  | Office Visits | 26,685 |  | 34,140 |  |
|  | ED Visits | 5,962 |  | 9,700 |  |
|  | Hospitalizations | 153 |  | 1,615 |  |
| Health Outcome | |  |  |  |  |
|  | Cases Prevented | 29,142 |  | 40,473 |  |
|  | Deaths Prevented | -172 |  | 562 |  |
| QALYs Gained (Undiscounted) | | | | | |
|  | Morbidity | 207 |  | 786 |  |
|  | Mortality | -2,106 |  | 8,519 |  |
|  | Total | -1,900 | TIIP Dominant | 9,305 | UIIP Dominant |
| QALYs Gained (Discounted 3%) | | | | | |
|  | Morbidity | 207 |  | 786 |  |
|  | Mortality | -1,334 |  | 4,833 |  |
|  | Total | -1,127 | TIIP Dominant | 5,619 | UIIP Dominant |
| QALYs Gained (Discounted 5%) | | | | | |
|  | Morbidity | 207 |  | 786 |  |
|  | Mortality | -1,085 |  | 3,755 |  |
|  | Total | -878 | TIIP Dominant | 4,541 | UIIP Dominant |
| Abbreviations: ED, emergency department; ICER, incremental cost-effectiveness ratio; $M, $ million; QALY, quality adjusted life years; TIIP, targeted influenza immunization program; UIIP, universal influenza immunization program  Note: An intervention is dominant if the intervention is more effective and less costly then the comparator. | | | | | |
