# Supplementary material for: Economic Appraisal of Ontario's Universal Influenza Immunization Program: A Cost-Utility Analysis
Source: PLoS Med. 2010 Apr 6;7(4):e1000256. doi: 10.1371/journal.pmed.1000256 (PMC2850382; doi:10.1371/journal.pmed.1000256)
Supplement: Table S7 — Results - sensitivity analysis using all respiratory conditions as outcome. (0.05 MB DOC) [file pmed.1000256.s009.doc]

| **Table S7:** Results – sensitivity analysis using all respiratory conditions as outcome | | | |
| --- | --- | --- | --- |
|  | **TIIP** | **UIIP** | **Incremental** |
| Immunization Program | $19,946,556 | $40,000,000 | $20,053,444 |
|  |  |  |  |
| Cost |  |  |  |
| Office visits | 14,040,030 | 3,618,948 | $10,421,081 |
| ED Visits | 13,781,009 | 4,113,765 | $9,667,244 |
| Hospitalizations | 21,115,453 | 13,249,973 | $7,865,480 |
| Total | 48,936,492 | 20,982,686 | $27,953,805 |
|  |  |  |  |
| Net Cost |  |  | -$7,900,361 |
|  |  |  |  |
| Resource Use |  |  |  |
| Office visits | 399,013 | 102,849 | 296,164 |
| ED Visits | 62,660 | 18,705 | 43,955 |
| Hospitalizations | 3,290 | 2,064 | 1,226 |
|  |  |  |  |
| Cases | 414,137 | 108,080 | 303,992 |
|  |  |  |  |
| Deaths | 647 | 393 | 254 |
|  |  |  |  |
| QALYs (undiscounted) | 11,882 | 5,309 | 6,573 |
| Morbidity | 6,586 | 1,859 | 4,726 |
| Mortality | 5,297 | 3,450 | 1,847 |
| QALYs (discounted 3%) | 9,647 | 3,966 | 5,682 |
| Morbidity | 6,586 | 1,859 | 4,726 |
| Mortality | 3,061 | 2,106 | 955 |
| QALYs (discounted 5%) | 9,026 | 3,564 | 5,463 |
| Morbidity | 6,586 | 1,859 | 4,726 |
| Mortality | 2,441 | 1,704 | 736 |
