# Supplementary material for: Economic Appraisal of Ontario's Universal Influenza Immunization Program: A Cost-Utility Analysis
Source: PLoS Med. 2010 Apr 6;7(4):e1000256. doi: 10.1371/journal.pmed.1000256 (PMC2850382; doi:10.1371/journal.pmed.1000256)
Supplement: Table S8 — Input parameter ranges. (0.04 MB DOC) [file pmed.1000256.s010.doc]

| Table S8: Input parameter ranges | | |
| --- | --- | --- |
| **Parameter** | **Range** | **Source** |
| *R_0_* | (1.2, 1.6) | [1,2] |
| *M* | (0.10, 0.20) | [3] |
| *e* | (0.6, 0.8) | [4] |
