# Supplementary material for: Economic Appraisal of Ontario's Universal Influenza Immunization Program: A Cost-Utility Analysis
Source: PLoS Med. 2010 Apr 6;7(4):e1000256. doi: 10.1371/journal.pmed.1000256 (PMC2850382; doi:10.1371/journal.pmed.1000256)
Supplement: Table S9 — Results of empirical versus predicted relative reduction. (0.03 MB DOC) [file pmed.1000256.s011.doc]

| Table S9: Results of empirical versus predicted relative reduction | | | | |
| --- | --- | --- | --- | --- |
|  | **Coverage** | | **Relative Reduction (mean, SD)** | |
|  | **1996/97** | **2005** | **Office visits**  **(Table 1)** | **Infection incidence**  **(model output,**  **filtered)** |
| Other provinces | 13% | 28% | 0.52  (0.51, 0.52) | 0.50  (0.48, 0.53) |
| Ontario | 18% | 42% | 0.21  (0.21, .022) | 0.19  (-0.03, 0.41) |
