# Supplementary material for: Economic Appraisal of Ontario's Universal Influenza Immunization Program: A Cost-Utility Analysis
Source: PLoS Med. 2010 Apr 6;7(4):e1000256. doi: 10.1371/journal.pmed.1000256 (PMC2850382; doi:10.1371/journal.pmed.1000256)
Supplement: Text S1 — Effectiveness of Ontario's UIIP. (0.06 MB DOC) [file pmed.1000256.s012.doc]

## Effectiveness of Ontario’s Universal Influenza Immunization Program (UIIP)

Note: This description is based on Kwong JC, Stukel TA, Lim J, McGeer AJ, Upshur RE, et al. (2008) The effect of universal influenza immunization on mortality and health care use. PLoS Med 5: e211.

The impact of Ontario’s UIIP on influenza-associated mortality, hospitalizations, and visits to emergency departments (EDs) and doctors’ offices was estimated using data from 1997 to 2004 (3 years before and 4 years after UIIP implementation). Influenza-associated outcomes were estimated by using multivariate regression models to generate baseline functions representing the hypothetical absence of influenza and then subtracting the expected baseline events from observed events during periods of influenza activity. The models controlled for age, sex, province, influenza surveillance data, and temporal trends.

### Estimation of baseline function

First, separate Poisson regression models were ran for each outcome, according to province and age group. Event counts were aggregated by week and sex within province-age group combinations. The dependent variable was the weekly event count for males and females, and the offset parameter was the province-age group-sex population. Models controlled for sex; viral surveillance for influenza A, influenza B, and RSV; the seasonal percentage of A(H3N2) isolates; the percentage of mismatched strains; linear and quadratic terms to model annual trends; and sine and cosine terms with periods of 1 y to model seasonal fluctuations, as in previous studies. To account for fluctuations in health service delivery during Christmas and post-Christmas holiday periods, categorical terms were included in the health care use models for these time periods. The expected baseline was generated by setting the influenza-related variables in the model (i.e., weekly percentage of tests positive for influenza A and B, seasonal percentage of A[H3N2] isolates) to zero as these were the terms that tracked influenza season peaks. Variance overdispersion was incorporated in the estimates of all standard errors (SE) to account for clustering of outcomes within weekly strata since outcomes to individuals within these strata may not be independent.

### Description of regression model

The multivariate Poisson regression model was expressed as follows:

ln(Y) = ln(population) + β0 + β1[sex] + β2[%FluA] + β3[%FluB] + β4[%RSV] + β5[%A(H3N2)] + β6[%mismatch] + β7[t] + β8[t2] + β9[sin(2tπ/52)] + β10[cos(2tπ/52)] + ε

Y represents the weekly number of events for a particular outcome (e.g., all-cause mortality) in a province for a specific age group and sex stratum. The offset term is the log of the annual province-, age- and sex-specific population size. β0 is the intercept and β1 estimates the effects of sex. β2 through β4 account for the weekly percentage of provincial specimens testing positive for influenza A, influenza B, and RSV, respectively. β5 accounts for the percentage of A(H3N2) isolates and β6 accounts for the percentage of circulating strains mismatched to vaccine strains in a season. β7 and β8 are the coefficients for the linear and quadratic time trend terms, with t expressed as the week since August 24, 1997 (1 to 416) divided by 52. β9 and β10 account for the seasonal cyclical pattern. We used a period of 1 year, as in previous studies. The error term ε represents random error in the model. Additional terms for Christmas holiday weeks and the post-Christmas holiday week were included in the health care use models to account for fluctuations in health care service delivery during holiday and post-holiday periods.

Weekly influenza-associated events were subsequently computed as the difference between the number of observed events and expected baseline events during periods of peak influenza activity, where expected counts were based on the adjusted Poisson models. These weekly estimates were aggregated to produce annual estimates. Overall and age-specific mean annual rates of influenza-associated outcomes were calculated for the periods before and after introduction of UIIP (pre-2000 versus post-2000), separately for Ontario and the other provinces combined.

Pre- and post-UIIP influenza-associated event rates were compared by dividing the adjusted postintervention rates by the preintervention rates to produce relative rates (RR) of UIIP effect separately for Ontario and the other provinces combined. The standard error of ln(RR) was computed assuming a Poisson distribution for the overall counts and incorporating the variance of the predicted baseline events. The pre-/post-RRs for Ontario and other provinces combined were compared using the z-test and expressed as a ratio.

Model fit was evaluated by examining the standardized Pearson residuals for outlying points and secular trends. We also evaluated the presence of influential provinces by removing them individually from the model and reestimating the UIIP effect. Although the model did not optimally fit the extreme, short-lived spikes that occurred during the peaks of the influenza season, the fit during the remainder of the season was reasonable. The Durbin-Watson *d*-statistic was used to test for autocorrelation in the residuals, both including and excluding these influenza season spikes, averaging the correlations across province- and age group-specific models. Analyses were performed using SAS 9.1 (SAS Institute). All statistical tests were computed at the 5% level of significance and were two sided.

Results of the regression analysis are presented in Table S6.
