# Supplementary material for: Economic Appraisal of Ontario's Universal Influenza Immunization Program: A Cost-Utility Analysis
Source: PLoS Med. 2010 Apr 6;7(4):e1000256. doi: 10.1371/journal.pmed.1000256 (PMC2850382; doi:10.1371/journal.pmed.1000256)
Supplement: Text S2 — Additional analysis using all respiratory conditions as outcome. (0.05 MB DOC) [file pmed.1000256.s013.doc]

## Additional analysis using all respiratory conditions as outcome

In the base case analysis, we used Pneumonia and Influenza diagnostic codes to extract the number of office visits, ED visits, and hospitalizations. Many influenza-related health care interactions are coded under different diagnostic codes (e.g., common cold, asthma, COPD, otitis media, etc.), we are therefore underestimating the health care resource use due to influenza. If we were to account for all cases seeking health care and cases not seeking health care, we would expect QALYs gained to increase and hence improve cost-effectiveness of the program.

In this additional analysis, we use the diagnostic codes for all respiratory conditions to extract health care utilization data. We find the program to be cost-saving (Table S7). While this still underestimates the true extent of influenza-related resource use, it is closer to what would be expected in terms of average attack rates, i.e. UIIP estimated number of cases (based on resource use) is 108,080. Taking into account that 67% of cases are symptomatic and 50% of those seeking health care, this would translate into 322,627 influenza infections in a population of 13 million, resulting in an attack rate of 2.5%. The attack rate based on the predicted resource use under a targeted immunization program (414,137) would have been 9.5%.
