# Supplementary material for: Economic Appraisal of Ontario's Universal Influenza Immunization Program: A Cost-Utility Analysis
Source: PLoS Med. 2010 Apr 6;7(4):e1000256. doi: 10.1371/journal.pmed.1000256 (PMC2850382; doi:10.1371/journal.pmed.1000256)
Supplement: Text S3 — Dynamic transmission model demonstrating herd immunity effects. (0.07 MB DOC) [file pmed.1000256.s014.doc]

## Dynamic transmission model demonstrating herd immunity effects

This analysis suggests that modestly improved vaccine coverage in Ontario provided significant benefits in terms of reduced numbers of events, relative to other provinces; according to Table S8, office visits dropped by 79% in Ontario after vaccine coverage changed from 18% to 42%. By comparison, office visits dropped by only 48% in other provinces when vaccine coverage changed from 13% to 28%. Such disproportionate increases from a 9% difference in coverage improvement may be attributable to herd immunity, which provides increasing incremental reductions in events as vaccine coverage increases. This should be especially true for influenza, which has a relatively low basic reproduction number.[1,2]

To test whether this is plausible, we analyzed a simple SIR compartmental model representing transmission during a single influenza season:

(A1)

We defined ranges for the initial proportion *M* of individuals immune due to exposure during previous influenza seasons or previous vaccines, the basic reproduction number *R*_0_, and vaccine effectiveness *e*. Previous immunity and vaccination in the current season modified the initial number of susceptible individuals according to

(A2)

where *p* is vaccine coverage. The intervals used appear in Table S8.

Dynamics in this region of parameter space are highly variable, with some parameter values giving small relative reductions while other parameter values from the same range giving very large relative reductions. Hence, we performed probabilistic uncertainty analysis as follows. We simulated the SIR model, sampling repeatedly and uniformly from the intervals in Table 1, and projected the reduction factor for the change in vaccine coverage estimated for other provinces (from 13% to 28%). The intervals came from published literature. We rejected any set of parameter values that did not result in a reduction factors in the range (0.47, 0.57), which is the mean empirical relative reduction for other provinces (0.52) plus or minus 5%. Sets of parameter values that survived this filter were then used to simulate the SIR model again, but now for the change in vaccine coverage observed in Ontario (from 18% to 42%) instead of the change observed in other provinces. The resulting mean and standard deviation of the relative reduction were tabulated.

The results of this analysis appear in Table S9. The mean (+/- 2SD) relative reduction for other provinces was 0.50 (0.48, 0.53), which, by design, is close to the empirical reduction because the filtering procedure discarded parameter sets that did not give rise to a relative reduction close to the empirical estimate. However, the corresponding mean (SD) relative reduction for Ontario, using the filtered parameter sets but the Ontario-specific change in vaccine coverage, was 0.19 (-0.03, 0.41). The mean relative reduction of 0.19 for Ontario is lower than the mean relative reduction of 0.52 for other provinces. A similar trend is observed in the empirical data. However, 59% of simulations using the Ontario change in coverage had a reduction factor of 0.21 or below. We note that the results vary somewhat depending on the intervals used, however, the intervals we use appear to be reasonable for influenza based on the existing literature.

While many outcomes are possible in this part of parameter space for the SIR model, and a more complex model with factors such as age structure and seasonality would be required to give a more definite answer, this basic analysis suggests that the results of Table 1 in the main text are not implausible.

The possibility of large relative reductions predicted for Ontario suggest that Ontario could be at or near a “tipping point” where modest increases in vaccine coverage result in disproportionate declines in infection prevalence and thus subsequent outcome such as physician office visits. To explore the possibility of a tipping point under UIIP, we investigated the filtered relative reduction for a wider range of vaccine coverage under the universal program. We plot the filtered relative reduction versus vaccine coverage in Figure S1, where relative reduction is measured in Ontario in 2005 relative to the situation in Ontario under 1996-7 coverage for a coverage of 18% as in Table S9 (thus, the horizontal axis representing coverage under UIIP ranges from 20% to 80%, including the baseline value of 42% that appears in Table S9). We also plot the corresponding proportion of simulations under the probabilistic uncertainty analysis that resulted in relative reductions below 0.01%, 0.1%, 1% and 5% in Figure S2. Figures S1 and S2 suggest a tipping point at approximately 40% vaccine coverage. Beyond this treshold value, the rate at which the relative reduction declines slows down (Figure S1) and the proportion of simulations where relative reduction falls below the threshold remains constant (Figure S2). This reflects the fact that, under the probabilistic uncertainty analysis, certain choices of parameter values make elimination of infection impossible because transmissibility is to high and/or vaccine efficacy is too low. Thus, some simulations still yield high values of the relative reduction, even for high vaccine coverage.

### References

1. Chowell G, Miller MA, Viboud C (2008) Seasonal influenza in the United States, France, and Australia: transmission and prospects for control. Epidemiol Infect 136: 852-864.

2. Coburn BJ, Wagner BG, Blower S (2009) Modeling influenza epidemics and pandemics: insights into the future of swine flu (H1N1). BMC Med 7: 30.

3. Ferguson NM, Mallett S, Jackson H, Roberts N, Ward P (2003) A population-dynamic model for evaluating the potential spread of drug-resistant influenza virus infections during community-based use of antivirals. J Antimicrob Chemother 51: 977-990.

4. Vu T, Farish S, Jenkins M, Kelly H (2002) A meta-analysis of effectiveness of influenza vaccine in persons aged 65 years and over living in the community. Vaccine 20: 1831-1836.
